# Preventive foot self-care practice and associated factors among diabetic patients attending the university of Gondar comprehensive specialized referral hospital, Northwest Ethiopia, 2021

**DOI:** 10.1186/s12902-022-01044-0

**Published:** 2022-05-11

**Authors:** Enyew Getaneh Mekonen, Tizita Gebeyehu Demssie

**Affiliations:** Department of Surgical Nursing, School of Nursing, College of Medicine and Health Sciences, University of Gondar, Gondar, Ethiopia

**Keywords:** Diabetic patients, Foot self-care, Practice, Northwest Ethiopia

## Abstract

**Background:**

Diabetes mellitus is emerging as a major worldwide health problem that has a social, financial, and developmental impact on developing countries. Foot complications are among the most serious and costly complications of diabetes which lead to lower extremity amputation due to diabetic foot ulcers. Poor diabetic foot self-care practice is identified by different studies as a major contributing factor to diabetic foot ulcers. Therefore, this study was intended to assess foot self-care practice and associated factors among diabetic patients attending the University of Gondar comprehensive specialized referral hospital.

**Methods:**

A hospital-based cross-sectional study was conducted from July 1 to August 30, 2021, at the University of Gondar comprehensive specialized referral hospital. A systematic random sampling technique was employed to select 384 diabetic patients. A structured pretested interviewer-administered questionnaire was used to collect data. The data was entered in epi-info version 7, analyzed using SPSS version 21, and presented using frequencies, percentages, tables, and graphs. Bivariable and multivariable analyses were investigated using a binary logistic regression model. *P*-value < 0.05 and an odds ratio with a 95% confidence interval were used to determine the significance and strength of the association.

**Results:**

Of the 384 diabetic patients, 46.4% (95% CI (41.1%-51.6%)) of them had poor foot self-care practice. Being male [AOR = 0.54, 95% CI (0.32, 0.89)], couldn’t read and write and completed primary education [AOR = 2.35, 95% CI (1.01, 5.43)] & [AOR = 2.92, 95% CI (1.39, 6.12)], living in rural area [AOR = 3.84, 95% CI (1.91, 7.75)], having diabetic complications [AOR = 2.19, 95% CI (1.07, 4.46)], taking both injection and pills [AOR = 0.33, 95% CI (0.12, 0.88)], having previous information about foot care [AOR = 0.12, 95% CI (0.06, 0.24)], and family support [AOR = 0.57, 95% CI (0.34, 0.94)] were determinants of poor foot self-care practice.

**Conclusion:**

The adherence of diabetic patients toward foot self-care practice was poor. Being male, having low educational status, living in a rural area, having diabetic-related complications, taking both injections and pills, not having previous information about foot care, and having poor family support increases the odds of having poor foot self-care practice. Giving health education to patients and their caregivers about the basic principles of diabetes foot care, like regular inspection of feet and appropriate footwear at their regular follow-up time, should be emphasized.

## Background

Diabetes mellitus (DM) is a group of metabolic diseases characterized by hyperglycemia which leads to peripheral neuropathy with a risk of foot ulcers and amputations as a long-term complication [1]. It is emerging as a major worldwide health problem that has a social, financial, and developmental impact, especially in low-and middle-income countries [2]. According to the 2017 International Diabetes Federation (IDF) study, there were 451 million (age 18–99 years) people with diabetes globally, which is expected to increase to 693 million by 2045 [3]. Ethiopia is one of the 48 countries in the IDF African region and more than 19 million people have diabetes in the region and it will be around 47 million by 2045 [4].

Foot complications are among the most serious and costly complications of DM, which end up with amputation of the lower extremity or part of it due to a foot ulcer [5]. Diabetic foot is one of the most significant complications of diabetes and is defined as a group of syndromes in which neuropathy, ischemia, and infection lead to tissue breakdown and possible amputation [6]. If a foot ulcer goes untreated and does not heal, it may become infected and 5–24% of foot ulcers will lead to limb amputation within six to eighteen months after the first evaluation [7, 8].

In the United States, more than 60% of non-traumatic lower extremity amputation (LEA) is done for diabetic patients with a rate of six to ten times higher than for people without diabetes. Nearly half of the patients required re-amputation within three to five years of the first LEA and approximately half of them died within five years after the amputation, with a higher risk for diabetic patients than non-diabetics [9].

Having diabetes is associated with an increased risk of amputation [10]. The overall amputation rate among patients with diabetic foot ulcers (DFU) in India was 28.4% [11]. Not having feet examined and not being given guidance on caring for feet at consultations during the previous year were associated with diabetic foot amputations [12]. Improper footwear was also considered a risk factor for amputation in patients with DFU [13].

In many African countries, where resources are already limited, diabetes imposes a heavy burden on already hostile health services and epidemiological surveys suggest that Africa has the second-highest worldwide prevalence of DFU among diabetics (7.2%) [14]. A study in Siri Lanka showed that even though more than half of the study participants had good knowledge of foot self-care principles, the practice was insufficient in that only regular foot inspection was performed by nearly two-thirds of them among all the principles of foot self-care [15].

According to the IDF Atlas, Ethiopia is one of Africa’s most populous countries with the highest number of people with diabetes [16]. The prevalence of diabetic foot ulcers was 14.8%, 12%, and 13.6% in studies conducted at Arba Minch hospital, Ayder referral hospital, and University of Gondar hospital, respectively and all of the studies identified poor diabetic foot self-care practice as the major factor associated with DFU [17–19]. Low education status, old age, and low awareness regarding diabetes were the risk factors for the poor practice of foot self-care [20].

A multi-disciplinary team approach, as well as regular comprehensive foot examination, patient education on foot care such as simple hygienic practices, provision of appropriate footwear, and prompt treatment of minor injuries, can reduce ulcer occurrence by half and amputations by up to 85% [8]. Since poor diabetic foot self-care practice is identified as the main factor for DFU which is associated with a high risk of LEA, this study is intended to assess foot self-care practice and associated factors among diabetic patients attending the University of Gondar comprehensive specialized referral hospital to supplement the efforts made in providing quality care and minimizing devastating consequences.

## Methods and materials

### Study design and period

An institutional-based cross-sectional study was conducted from July 1 to August 30, 2021.

### Study area

The study was conducted at the University of Gondar comprehensive specialized referral hospital, which is the only Comprehensive Specialized Referral hospital located in Gondar city. Gondar city is located in the Central Gondar administrative zone, which is about 727 km away from Addis Ababa, the capital of Ethiopia, and 180 km away from Bahir Dar, the capital city of Amhara National Regional State. The University of Gondar comprehensive specialized referral hospital is a referral teaching hospital with 400 beds that provides service to more than five million people. Continuous care for chronic illnesses, including diabetes mellitus, is one of the many services rendered by the hospital.

### Source and study populations

All diabetic patients who attend the diabetic follow-up clinic of the University of Gondar comprehensive specialized referral hospital were considered as source populations of the study. All diabetic patients who attended the diabetic follow-up clinic of the University of Gondar comprehensive specialized referral hospital during the study period were the study population.

### Inclusion and exclusion criteria

All diabetic patients who attended the diabetic follow-up clinic of the University of Gondar referral hospital during the study period were included in the study. Patients who were seriously ill and unconscious were excluded from the study.

### Sample size determination and sampling procedure

The sample size was computed using a single population proportion formula by taking the proportion of foot self-care practice at 39.0% (36), 95% confidence interval, and a 5% margin of error. The final sample size was 384 after adding a 5% non-response rate. A systematic random sampling technique was employed to recruit the required participants for the study. Study participants were selected by calculating the “k” value from the total estimated population. The total estimated population in the two-month data collection period was 1500, according to the information taken from the office of the chronic disease follow-up clinic. Then k = 1500/384 = 4; so, the study participants were selected at 4 intervals until the calculated sample size was attained.

### Variables of the study

Foot self-care practice (good/poor) is the dependent variable.

Independent Variables: socio-demographic characteristics (age, sex, marital status, educational status, occupation, and residence); clinical factors (type of DM, duration of DM, family history of DM, comorbidity, diabetic complications, history of foot ulcer, type of medication); personal factors (family support).

### Operational definitions

Comorbidity: A diabetic patient who had a known additional disease other than DM was considered as having comorbidity [21].

Diabetic complications: A diabetic patient who had one of the following (retinopathy, nephropathy, neuropathy, myocardial infarction, and stroke) by reviewing the patient chart was considered to have diabetes complications [22].

Family support: participants who scored mean and above the family adaptation, partnership, growth, affection, and resolve (APGAR) score were considered to have good family support, while those who scored below the mean were categorized to have poor family support.

Good foot self-care practice: participants who scored mean or above on practice related-questions were considered to have a good practice.

Poor foot self-care practice: participants who scored below the mean on practice related-questions were considered to have poor practice.

### Data collection instruments and procedures

Data was collected using a structured pre-tested questionnaire adapted from previous studies [23, 24] via face-to-face interviews. The questionnaire contained 38 questions arranged in four dimensions: seven socio-demographic questions, nine clinical-related questions, seventeen-foot self-care practice-related questions, and five questions to assess family support. Foot self-care practice was assessed by using a questionnaire adapted from a validated tool of the Nottingham Assessment of Functional Foot Care revised 2015 (NAFFC) [25]. The tool was proven to be valid and reliable for assessing diabetic foot care behavior [26]. Each question was responded to on a scale ranging from 0 to 3 according to the frequency of existence of the practice. This study used only 17 items out of the 26 questions of the 2015 revised NAFFC since the socioeconomic status of participants in the study area is different. Family support was measured using the family APGAR (adaptation, partnership, growth, affection, and resolve) scale which consists of 5 items, scored from 0 (hardly ever) to 2 (almost always) [27]. The total score range is from 0 to 10. The larger the score, the greater the amount of satisfaction with family functioning. The Cronbach’s alpha of the subscale was 0.86 [28].

### Data processing and analysis

Following data collection, each questionnaire was reviewed for completeness and consistency and possible corrections were done by investigators. Data was entered into Epi-info version 7 and transferred into SPSS version 21 and then, data cleaning and coding were done to make it ready for analysis. The results of the descriptive statistics were expressed as mean, standard deviation, percentage, and frequency using tables, and graphs. Binary logistic regression was employed to identify factors associated with foot care practice. Those variables with a *p*-value less than or equal to 0.2 from the bivariable analysis were a candidate for multivariable analysis. The multivariable analysis was used to control for potential confounders and a *p*-value of < 0.05 was used to declare the significance of the association. Moreover, the strength of the association between different independent variables with the dependent variable was measured using odds ratios with a 95% confidence interval.

### Management of data quality

The data collection instrument was prepared in English and translated into the local language, Amharic, and back-translated to English by language experts to check for consistency. A pretest was done on 5% of the total sample size at Debre Tabor referral hospital. Necessary modifications were made upon the identification of ambiguity in the questionnaire. We recruited, trained, and assigned three diploma nurses and one MSc nurse for data collection and supervision, respectively. The one-day training was given to both the data collectors and supervisor about the objective of the study, the technique of data collection, the content of the questionnaire, and the issue of confidentiality of the participants.

## Results

### Socio-demographic and clinical characteristics of diabetic patients

A total of 384 diabetic patients participated in this study, with a 100% response rate. The mean age of the respondents was 49.80 ± 16.80 SD years and 19.3% of them fall in the age range of 49–58 years. More than half (50.5%) of the diabetic patients were male and 58.3% of them were married. Regarding their educational status, more than one-third (34.6%) of the participants have completed primary (grade 1–8) education. Concerning their religion, more than two-thirds (68.2%) of the diabetic patients were Orthodox Tewahido religion followers. Ninety-eight (25.5%) of the study participants were merchants followed by a gov't employee (17.7%). The majority (79.2%) of the respondents were urban dwellers (Table 1).

**Table 1:** Socio-demographic characteristics of diabetic patients at the University of Gondar comprehensive specialized referral hospital, northwest Ethiopia, 2021 (*n* = 384)

Variables	Category	Frequency (*n* = 384)	Percentage (100%)
Age (in years)	19–28	65	16.9
	29–38	41	10.7
	39–48	71	18.5
	49–58	74	19.3
	59–68	79	20.6
	≥ 69	54	14.0
Sex	Female	190	49.5
	Male	194	50.5
Marital Status	Single	66	17.2
	Married	224	58.3
	Widowed	51	13.3
	Divorced	43	11.2
Educational status	Can’t read and write	93	24.2
	Primary (grade 1–8)	133	34.6
	Secondary (grade 9–12)	86	22.4
	College and above	72	18.8
Religion	Orthodox	262	68.2
	Muslim	99	25.8
	Protestant	17	4.4
	Others^a^	6	1.6
Occupation	Farmer	61	15.9
	Merchant	98	25.5
	Gov't employee	68	17.7
	Private employee	56	14.6
	Student	34	8.9
	Others^b^	67	17.4
Residence	Rural	80	20.8
	Urban	304	79.2

^a^Catholic

^b^Retired, Housewife, Daily laborer

One hundred and fifty-nine (41.4%) of the respondents didn’t know the type of DM they had been diagnosed with. More than three-fourths (78.4%) of diabetic patients had not a family history of DM. More than half (56.0%) of the study participants lived with DM for five years and below. Nearly one-fifth (18.8%) of the respondents had comorbidity, of which three-fourths (75%) of them had hypertension. Only fifty (13.0%) of the study participants had diabetic complications, of which 38.0% of them had nephropathy. The majority (87.2%) of diabetic patients did not have a history of diabetic foot ulcers. Regarding the type of medication, one hundred and eighty-two (47.4%) of the respondents used injections only (Table 2).

**Table 2:** Clinical-related characteristics of diabetic patients at the University of Gondar comprehensive specialized referral hospital, northwest Ethiopia, 2021 (*n* = 384)

Variables	Category	Frequency (*n* = 384)	Percentage (100%)
Type of DM	Type one	106	27.6
	Type two	119	31.0
	Not sure	159	41.4
Family history of DM	Yes	83	21.6
	No	301	78.4
Duration of DM	≤ 5 years	215	56.0
	6–10 years	122	31.8
	11–15 years	30	7.8
	≥ 16 years	17	4.4
Comorbidity	Yes	72	18.8
	No	312	81.2
Type of comorbidity (*n* = 72)	Hypertension	54	75.0
	Heart failure	6	8.3
	Asthma	5	7.0
	Others^a^	7	9.7
Complications	Yes	50	13.0
	No	334	87.0
Type of complication (*n* = 50)	Nephropathy	19	38.0
	Neuropathy	15	30.0
	Retinopathy	15	30.0
	Cardiovascular disease	1	2.0
History of foot ulcer	Yes	49	12.8
	No	335	87.2
Type of medication	Injection only	182	47.4
	Pills only	171	44.5
	Both injection and pills	31	8.1

^a^HIV/AIDS, skin infection, renal stone

*DM* Diabetes Mellitus

### Diabetic patients’ foot self-care practice

**Fig. 1:**
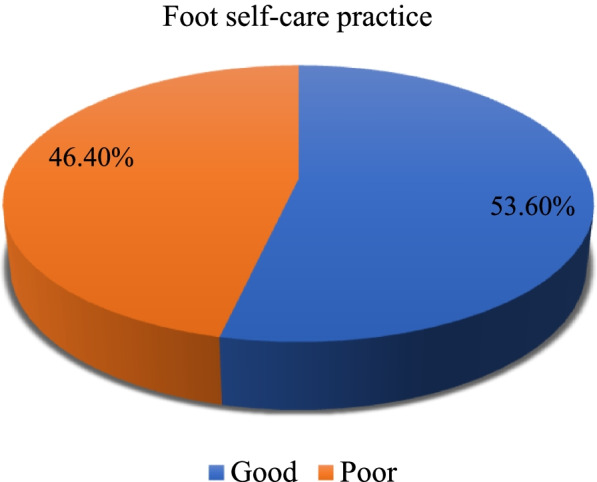
Foot self-care practice among diabetic patients attending the University of Gondar comprehensive specialized referral hospital, northwest Ethiopia, 2021 (*n* = 384)

Of the 384 diabetic patients, 46.4% (95% CI (41.1-51.6%)) of them had poor foot self-care practice (Fig. 1). More than one-third (35.4%) of diabetic patients examined their feet once a day and 45.8% of them often checked their shoes before they put them on. One hundred and fifty-one (39.3%) of the participants never checked their shoes when they took them off. More than half (56.2%), 40.9, and 45.3% of the respondents washed their feet once a day, often checked their feet were dry after washing, and rarely/never dried between their toes, respectively. Two hundred twenty and eight (59.4%) diabetic patients never used moisturizing cream on their feet and more than two-thirds (69.8%) of them never put moisturizing cream between their toes. One hundred seventy (44.3%) of the participants cut their toenails about once a month and nearly half (50.5%) of them sometimes wore slippers with no fastening. More than one-third (38.5 and 35.4%) of the respondents sometimes wore shoes without socks/stockings/tights and changed their socks/stockings/tights daily, respectively. More than half (53.6%) and the majority (90.0%) of diabetic patients never walked around the house or outside on bare feet, respectively. More than two-fourths (77.9%) of the respondents never put their feet near the fire. Two hundred thirty and seven (61.7%) and 62.0% of the study participants never put a dry dressing on a blister and a graze, cut, or burn when they get one respectively (Table 3).

**Table 3:** Diabetic patients’ foot self-care practice at the University of Gondar comprehensive specialized referral hospital, northwest Ethiopia, 2021 (*n* = 384)

No	Questions	Responses (frequency (percentage))
1	Do you examine your feet?	Once a week or less (103 (26.8%))
		2–6 times a week (94 (24.5%))
		Once a day (136 (35.4%))
		More than once a day (51 (13.3%))
2	Do you check your shoes before you put them on?	Never (58 (15.1%)) Rarely (84 (24.9%))
		Sometimes (66 (17.2%)) Often (176 (45.8%))
3	Do you check your shoes when you take them off?	Never (151 (39.3%)) Rarely (77 (20.1%))
		Sometimes (67 (17.4%)) Often (89 (23.2%))
4	Do you wash your feet?	A few days a week (8 (2.1%))
		Most days a week (28 (7.3%))
		Once a day (216 (56.2%))
		More than once a day (132 (34.4%))
5	Do you check your feet are dry after washing?	Never (73 (19.0%)) Rarely (80 (20.8%))
		Sometimes (74 (19.3%)) Often (157 (40.9%))
6	Do you dry between your toes?	Rarely/Never (174 (45.3%) Often (34 (8.9%))
		Sometimes (93 (24.2%)) Always (83 (21.6%))
7	Do you use moisturizing cream on your feet?	Never (228 (59.4%))
		About once a month (22 (5.7%))
		Once a week (43 (11.2%)) Daily (91 (23.7%))
8	Do you put moisturizing cream between your toes?	Daily (59 (15.4%))
		Never (268 (69.8%))
		About once a week (37 (9.6%))
		About once a month (20 (5.2%))
9	Are your toenails cut?	Never (7 (1.8%))
		Less than once a month (98 (25.5%))
		About once a month (170 (44.3%))
		About once a week (109 (28.4%))
10	Do you wear slippers with no fastening?	Most of the time (41 (10.7%))
		Sometimes (194 (50.5%))
		Rarely (96 (25.0%))
		Never (53 (13.8%))
11	Do you wear shoes without socks/stockings/tights?	Often (80 (20.8%))
		Sometimes (148 (38.5%))
		Rarely (90 (23.4%))
		Never (66 (17.2%))
12	Do you change your socks/stockings/tights?	Less than 4 times a week (116 (30.2%))
		4–6 times a week (121 (31.5%))
		Daily (136 (35.4%))
		More than once a day (11 (2.9%))
13	Do you walk around the house on bare feet?	Often (24 (6.3%))
		Sometimes (114 (29.7%))
		Rarely (40 (10.4%)
		Never (206 (53.6%))
14	Do you walk outside on bare feet?	Often (6 (1.6%))
		Sometimes (16 (4.2%))
		Rarely (16 (4.2%))
		Never (346 (90.0%))
15	Do you put your feet near the fire?	Often (4 (1.0%))
		Sometimes (52 (13.5%))
		Rarely (29 (7.6%))
		Never (299 (77.9%))
16	Do you put a dry dressing on a blister when you get one?	Never (237 (61.7%))
		Rarely (72 (18.8%))
		Sometimes (56 (14.6%))
		Often (19 (4.9%))
17	Do you put a dry dressing on a graze, cut, or burn when you get one?	Never (238 (62.0%))
		Rarely (60 (15.6%))
		Sometimes (62 (16.1%))
		Often (24(6.3%))

### Factors associated with foot self-care practice

In bivariable logistic regression analysis, variables like age, marital status, sex, educational status, residence, diabetic complications, history of foot ulcer, type of medication, having information about foot self-care practice, and family support were competent for multivariable analysis. Sex, educational status, residence, diabetic complications, type of medication, having information about foot self-care practice, and family support were statistically significant factors associated with foot self-care practice in multivariable logistic regression analysis.

Female diabetic patients were 54% less likely to have poor foot self-care practice than males [AOR = 0.54, 95% CI (0.32, 0.89)]. Diabetic patients who couldn’t read and write and completed primary education were nearly two and three times more likely to have poor foot self-care practice compared with those who completed college and above education respectively [AOR = 2.35, 95% CI (1.01, 5.43)] and [AOR = 2.92, 95% CI (1.39, 6.12)]. Diabetic patients who come from rural areas were nearly four times more likely to have poor foot self-care practice than those who come from urban areas [AOR = 3.84, 95% CI (1.91, 7.75)]. The odds of having poor foot self-care practice were nearly two times higher among patients with diabetic complications compared with their counterparts [AOR = 2.19, 95% CI (1.07, 4.46)]. Diabetic patients who used injections only were 33% less likely to have poor foot self-care practice compared with patients who took both injections and pills [AOR = 0.33, 95% CI (0.12, 0.88)]. Those diabetic patients who had no previous information about foot self-care practice were 88% times more likely to have poor foot self-care practice than patients with previous information [AOR = 0.12, 95% CI (0.06, 0.24)]. Diabetic patients who had good family support were 57% less likely to have poor foot self-care practice compared with patients with poor family support [AOR = 0.57, 95% CI (0.34, 0.94)] (Table 4).

**Table 4:** Bivariable and multivariable logistic regression analysis of factors associated with foot self-care practice among diabetic patients attending the University of Gondar comprehensive specialized referral hospital, northwest Ethiopia, 2021 (*n* = 384)

Variables		Foot self-care practice		OR with 95% CI		*P*-value
		**Poor**	**Good**	**Crude**	**Adjusted**	
Age (in years)	≥ 69	28	26	1.62 (0.78, 3.35)	0.93 (0.33, 2.61)	0.882
	59–68	29	50	0.87 (0.44, 1.71)	1.004 (0.42, 2.39)	0.993
	49–58	41	33	1.86 (0.95, 3.66)	1.02 (0.41, 2.54)	0.963
	39–48	34	37	1.38 (0.70, 2.72)	0.42 (0.16, 1.08)	0.072
	29–38	20	21	1.43 (0.65, 3.14)	0.56 (0.19, 1.62)	0.281
	19–28	26	39	1	1	
Sex	Female	76	114	0.60 (0.40, 0.90)	0.54 (0.32, 0.89)*	0.016
	Male	102	92	1	1	
Educational status	Can’t read and write	55	38	5.07 (2.53, 10.13)	2.35 (1.01, 5.43)*	0.046
	Primary (grade 1–8)	78	55	4.96 (2.58, 9.55)	2.92 (1.39, 6.12)*	0.005
	Secondary (grade 9–12)	29	57	1.71 (0.87, 3.63)	1.05 (0.46, 2.39)	0.903
	College and above	16	56	1	1	
Marital Status	Single	28	38	1.14 (0.54, 2.41)	1.22 (0.31, 4.73)	0.778
	Married	106	118	1.39 (0.75, 2.59)	1.70 (0.70, 4.14)	0.245
	Widowed	24	19	1.96 (0.86, 4.46)	2.18 (0.75,6.34)	0.153
	Divorced	20	31	1	1	
Residence	Rural	65	15	7.32 (3.99, 13.45)	3.84 (1.91, 7.75)*	< 0.001
	Urban	113	191	1	1	
Complications	Yes	32	18	2.23 (1.24, 4.24)	2.19 (1.07, 4.46)*	0.031
	No	146	188	1	1	
History of foot ulcer	Yes	30	19	1.99 (1.08, 3.69)	1.21 (0.53, 2.76)	0.653
	No	148	187	1	1	
Type of Medication	Pills only	69	102	0.28 (0.12, 0.64)	0.53 (0.20, 1.40)	0.197
	Injection only	87	95	0.38 (0.16, 0.86)	0.33 (0.12, 0.88)*	0.027
	Both injection and pills	22	9	1	1	
Having information	Yes	107	193	0.10 (0.05, 0.19)	0.12 (0.06, 0.24)*	< 0.001
	No	71	13	1	1	
Family support	Good	57	106	0.44 (0.29, 0.67)	0.57 (0.34, 0.94)*	0.028
	Poor	121	100	1	1	

^*^Statistically significant at *p*-value < 0.05

*CI* Confidence Interval, *OR* Odds Ratio

## Discussion

Foot self-care practice is one of the most important self-management behaviors to prevent the occurrence of diabetic foot ulcers. Preventing foot complications must be emphasized because foot ulcers of neuropathic origin are highly preventable and prevention is better than cure. In the current study, 46.4% (95% CI [41.1-51.6%)] of diabetic patients had poor foot self-care practice. This finding was in agreement with studies conducted in Bahir Dar, Ethiopia (47.0%), Kenya (51.2%), and Guilan Province (north of Iran) (49.6%) [23, 29, 30]. However, this finding was higher than studies conducted in Hawassa, Ethiopia (34.8%), Pakistan (39.8%), and Baquba city, Iraq (37.5%) [31–33]. The possible justification for this difference might be due to the difference in study participants and data collection tools. The previous studies were conducted among patients with type-2 diabetes, while the current study used data from patients with both types of diabetes. The study conducted in Hawassa has used the summary of diabetes self-care activities tool (15 items) to measure the five domains of diabetes self-care practices (diet, exercise, medication, foot care, and self-monitoring of blood glucose), of which foot care practice was measured by only five questions which might exaggerate the level of practice. The current study, on the other hand, used the Nottingham assessment of functional foot care, which is a valid and reliable tool to assess diabetic foot care practice. This might also be due to the difference in the socioeconomic status of the participants in Pakistan, Iraq, and Ethiopia.

On the other hand, the current finding was lower than studies conducted in Dessie Referral Hospital, Ethiopia (61%), Egypt (62.2%), Vietnam (58.6%), India (58.4%), Kuwait (69.2%), Kuantan, Malaysia (59.6%), another study in India (83.4%), Jinnah Hospital, Lahore (86%), Chinese General Hospital and Medical Center (77.6%), Malaysia (61.8%), and Rural Chennai (58.6%) [24, 34–43]. The plausible justification for this difference might be due to the difference in data collection tool, the difference in study participants (most of the previous studies were conducted among type 2 diabetic patients), the difference in sampling technique (a non-probability convenience sampling technique was utilized in Lahore’s and Malaysia’s studies), and study setting (the study conducted in Rural Chennai was conducted in a rural health center, while the current study was conducted in a comprehensive specialized referral hospital in which most diabetic patients might have good adherence to foot self-care practices).

Male diabetic patients were more likely to have poor foot self-care practice than females. This finding was supported by studies conducted in Dessie Referral Hospital, Ethiopia, Turkey, and Canada [24, 44, 45]. This might be attributed to women more habitually performing the care essential to prevent ulcerations, like drying between the toes after washing, regular foot checking, appropriately trimming nails to avoid lesions, not walking barefoot, and performing good hygiene to decrease the risk of infections [46]. Diabetic patients who couldn’t read and write and completed primary education were nearly two and three times more likely to have poor foot self-care practice compared with those who completed college and above education respectively. Similar findings were reported by studies conducted in Bahir Dar, Ethiopia, Kenya, Egypt, India, Vietnam, Indonesia, Jinnah Hospital, Lahore, and Baquba city, Iraq [23, 30, 33, 34, 37, 38, 41, 42, 47]. This could be because as a diabetic patient’s educational status improves, so will his or her awareness of foot self-care principles, which is the fundamental preventive measure of diabetic foot ulceration, which will be practiced regularly. This might also be attributed to individuals with a higher educational status being expected to read and obtain more information regarding their disease and foot self-care as well as realize the evidence they obtained from their caregivers. Health-related knowledge among less-educated individuals is lower, which leads to unhealthy activities compared with those who have higher educational status [48].

Diabetic patients living in rural areas were nearly four times more likely to have poor foot self-care practice than those who live in an urban area. Studies conducted in Dessie Referral Hospital, Ethiopia, and Guilan Province (north of Iran) reported similar findings [24, 29]. This might be due to diabetic patients living in rural areas having less access to information through health education about self-care practices, books, and social media than patients living in urban areas. The odds of having poor foot self-care practice were nearly two times higher among patients with diabetic complications compared with their counterparts. This finding was supported by studies conducted in Guilan Province (north of Iran) and Turkey [29, 45]. This might be due to patients with diabetic complications, specifically diabetic retinopathy, having visual deterioration or blindness which minimizes their ability to examine and take care of their feet. Diabetes patients with retinopathy were at considerable risk of developing diabetic foot ulceration due to severe loss of vision or blindness [49].

Diabetic patients who took both injections and pills were more likely to have poor foot self-care practice compared with patients who used injections only. Similar findings were reported by studies conducted in Kuwait and India [35, 41]. This might be attributed to the medication burden which hinders their ability to adhere to self-care behaviors and their readiness to integrate foot self-care practice into their daily lives. Those diabetic patients who had no previous information about foot self-care practice were more likely to have poor foot self-care practice than patients with previous information. Studies conducted in Hawassa, Ethiopia, and Guilan Province (north of Iran) reported similar findings [29, 31]. This might be due to diabetic patients who get information and are advised to take care of their feet by health professionals who tend to have good foot self-care practice [50]. Educating patients and their families regarding proper management and care of their feet is very important to increase patients’ self-care capacity, which in turn minimizes the risk of developing diabetic foot ulcers [51]. Diabetic patients who had poor family support were more likely to have poor foot self-care practice compared with patients with good family support. This finding is in agreement with a study conducted in Indonesia [47]. This might be attributed to the fact that the family affects the management of patients with chronic illness [52]. Good support from friends and family encourages adherence to foot self-care practice by inspiring optimism and self-esteem.

This study has some limitations. First, the study was hospital-based, which might not represent the true picture of foot self-care practice in the community. Secondly, the study did not incorporate health professional-related factors that affect foot self-care practice. Also, the cross-sectional nature of the study design makes it difficult to establish cause and effect relationships.

## Conclusion

The adherence of diabetic patients toward foot self-care practice was poor. Being male, having low educational status, living in a rural area, having diabetic-related complications, taking both injections and pills, not having previous information about foot care, and poor family support increase the odds of having poor foot self-care practice among diabetic patients. It is better to give health education to patients and their caregivers about the basic principles of diabetes foot care, like regular inspection of the feet and appropriate footwear, as well as how to encourage patients to adhere to foot self-care practice at their regular follow-up time. Health professionals are expected to give special attention to male patients, patients with low educational levels, who come from rural areas, who have diabetic complications, and who take both insulin and pills at the time of health education.

## Data Availability

All data is available upon request. The reader could contact the corresponding author for the underlying data.

## Abbreviations

AOR: Adjusted Odds Ratio
CI: Confidence Interval
DFU: Diabetic Foot Ulcer
DM: Diabetes Mellitus
IDF: International Diabetes Federation
LEA: Lower Extremity Amputation
NAFFC: Nottingham Assessment of Functional Foot Care
SD: Standard Deviation
SPSS: Statistical Product and Service Solutions
